# Supplementary material for: GCNA is a histone binding protein required for spermatogonial stem cell maintenance
Source: Nucleic Acids Res. 2023 Mar 15;51(10):4791–813. doi: 10.1093/nar/gkad168 (PMC10250205; doi:10.1093/nar/gkad168)
Supplement: gkad168_Supplemental_File [file gkad168_supplemental_file.pdf]

Supplementary Figure Legends

Supplementary Figure S1. Generation and validation of GCNA-deficient mice.

(A) Schematic representation of the different alleles of Gcna used for this study. Coding regions in exons are displayed in white boxes and non-coding are displayed in black boxes. Primers and PCR fragments lengths used for genotyping are highlighted in red. LoxP sites are highlighted in green. The region recognised by two antibodies directed against mouse GCNA (Tra98, GCNA-1) is highlighted in blue.

(B) Endpoint genotyping PCRs obtained using the PCR primers showed in (A).

(C) Validation of *Gcna*$^{-}$/Y mice by immunofluorescence of testis sections, using the Tra98 antibody. LAMIN B1 was displayed in red, Tra98 in green and DNA (DAPI stained) in blue.

Supplementary Figure S2. Phenotyping of GCNA-deficient male mice.

(A) Schematic representation of the breeding strategy to obtain wild-type or *Gcna*$^{-}$/Y males and wild-type or heterozygous females mice.

(B) Expected and observed frequencies of each genotypes obtained from the breeding strategy presented in (A).

(C) Representative photograph of 1 year old wild-type and *Gcna*$^{-}$/Y male mice.

(D) Kaplan-Meier curves of the survival of wild-type and *Gcna*$^{-}$/Y male mice up to 1 year old. 1.5 months WT (n=6 mice) and *Gcna*$^{-}$/Y (n=5 mice), 3 months WT (n=8 mice) and *Gcna*$^{-}$/Y (n=6 mice), 6 months WT (n=7 mice) and *Gcna*$^{-}$/Y (n=6 mice), 12 months WT (n=9 mice) and *Gcna*$^{-}$/Y (n=6 mice).

(E) Analysis of GCNA expression in different adult mice tissues by Western blot. Twenty-five micrograms of proteins were loaded per well. Blots were probed with two antibodies directed against mouse GCNA (Tra98 and GCNA-1) and with an anti-beta-actin antibody.

(F) Seminal vesicle weight of wild-type and *Gcna*$^{-}$/Y male mice between 1.5 months and 12 months. 1.5 months WT (n=6 mice) and *Gcna*$^{-}$/Y (n=5 mice), 3 months WT (n=6 mice) and *Gcna*$^{-}$/Y (n=4 mice), 6 months WT (n=7 mice) and *Gcna*$^{-}$/Y (n=6 mice), 12 months WT (n=8 mice) and *Gcna*$^{-}$/Y (n=5 mice). Data represent the mean and S.D..

(G) Testosterone concentration in serum of 1 year old wild-type (n=7) and *Gcna*$^{-}$/Y (n=5) mice. Data represent the mean and S.D.. *P* value was calculated by using an unpaired t-test.

(H) Representative micrographs of DAPI stained epididymis sections of 6 weeks old wild-type and *Gcna*$^{-}$/Y male mice.

(I) Assessment of the fertility of wild-type and *Gcna*$^{-}$/Y male mice. Wild-type and *Gcna*$^{-}$/Y male mice were bred with wild-type females and the number of alive pups were counted after birth. Mice were set up at 7 weeks and remained with females until 23 weeks old. Wild-type (n=3 mice, 13 litters) and *Gcna*$^{-}$/Y (n=3 mice, 12 litters). Data represent the mean and S.D.. *P* value was calculated by using an unpaired t-test.

Supplementary Figure S3. Fertility of GCNA-deficient female mice.

(**A**) Schematic representation of the breeding strategy to obtain wild-type or *Gcna*$^{-}$/Y males and heterozygous and *Gcna*$^{-/-}$ females mice.

(**B**) Expected and observed frequencies of each genotypes obtained from the breeding strategy presented in (A).

(**C**) Micrographs of Haematoxylin and Eosin stained ovaries sections of 2 and 6 months old wild-type and *Gcna*$^{-/-}$ mice.

(**D**) Quantification of follicles per ovaries section of 2 and 6 months old mice. Two months WT (n=3 mice) and *Gcna*$^{-/-}$ (n=4 mice), 6 months WT (n=5 mice) and *Gcna*$^{-/-}$ (n=4 mice). Data represent the mean and S.D.. *P* values were calculated by using an unpaired t-test.

(**E**) Assessment of the fertility of wild-type and *Gcna*$^{-/-}$ female mice. Wild-type and *Gcna*$^{-/-}$ female mice were bred with wild-type males and the number of alive pups were counted after birth. Wild-type (n=3 mice, 13 litters) and *Gcna*$^{-/-}$ (n=3 mice, 11 litters). Data represent the mean and S.D.. *P* values were calculated by using an unpaired t-test.

(**F**) *Gcna*$^{-/-}$ female mice were bred with wild-type males and pups were genotyped after birth.

(**G**) Expected and observed frequencies of each genotypes obtained from the breeding strategy presented in (F).

Supplementary Figure S4. GCNA is dispensable for male PGC development.

(A) Quantification of GCNA mRNA expression in PGCs (GOF18-GFP positive cells) from male embryos at E11.5 and E12.5 and in the testis from postnatal day 2 to day 21.

(B) Schematic representation of the breeding strategy to obtain wild-type or *Gcna*$^{-}$/Y male carrying the GOF18-GFP PGC reporter.

(C) Micrographs of GOF18-GFP fluorescence in E12.5 male gonads from wild-type and *Gcna*$^{-}$/Y embryos carrying the GOF18-GFP PGC reporter.

(D) Representative flow cytometry plots (SSEA1/GOF18-GFP) from E12.5 wild-type and *Gcna*$^{-}$/Y male embryos.

(E) Quantification of PGCs (SSEA1 and GOF18-GFP double positive cells) from male embryos at E12.5 (wild-type, n=7 embryos and *Gcna*$^{-}$/Y, n=4 embryos). Data represent the mean and S.D.. P value was calculated by using an unpaired t-test.

Supplementary Figure S5. GCNA is required for maintenance of PLZF+ cells in mouse

(A) Analysis of PLZF and WT1 expression in testes of 10 weeks old mice by Western blot. Twenty-five micrograms of proteins per wells were loaded. Blot was probed with antibodies directed against mouse GCNA (GCNA-1), PLZF, WT1 and with an anti-LAMIN B1 antibody.

(B) Quantification of the PLZF signal in (A) relative to LAMIN B1 (wild-type, n=6 mice and *Gcna*$^{-}$/Y, n=6 mice). Data represent the mean and S.D.. *P* value was calculated by using an unpaired t-test.

(C) Quantification of the WT1 signal in (A) relative to LAMIN B1 (wild-type, n=6 mice and *Gcna*$^{-}$/Y, n=6 mice). Data represent the mean and S.D.. *P* value was calculated by using an unpaired t-test

(D) Immunofluorescence staining of Plzf-tdTomato on testis sections of 6 months old wild-type and *Gcna*$^{-}$/Y mice.

(E) Right, quantification of the number of Plzf-tdTomato positive cells per seminiferous tube in wild-type and *Gcna*$^{-}$/Y testes. Left, quantification of the frequency of seminiferous tube with no Plzf-tdTomato positive cells in wild-type and *Gcna*$^{-}$/Y testes. Data represent mean and S.D., n=4 six month old mice per group.

Supplementary Figure S6. USGs are not exhibiting increased apoptosis nor enhanced differentiation in absence of GCNA.

(A) Immunofluorescence staining of cleaved caspase 3 (CC3) on testis sections of 6 months old wild-type and *Gcna*$^{-}$/Y mice.

(B) Average number of CC3 positive cells per seminiferous tubes at 6 months old. At least 50 seminiferous tubes are scored per mouse. Data represent the mean and S.D. (n=3 mice for each genotype). alues ere calculated using an unpaired t test.

(C) Frequency of seminiferous tubes that are not exhibiting any CC3 positive cells in 6 months old mice. At least 50 seminiferous tubes are scored per mouse. Data represent the mean and S.D. (n=3 mice for each genotype). alues ere calculated using an unpaired t test.

(**D**) Immunofluorescence staining of wild-type and *Gcna*$^{-}$/Y testis sections from 6 months old mice. Cleaved Caspase 3 (CC3) was displayed in red, PLZF in green and DNA (DAPI stained) in blue. White arrows highlight PLZF positive USGs and yellow arrows highlight CC3 positive germ cells.

(**E**) Frequency of PLZF positive cells exhibiting a CC3 positive signal at 1.5 and 6 months old. A minimum of 50 PLZF positive cells are scored per mouse. Data represent the mean and S.D. (n=3 mice for each genotype and age).

(F) Immunofluorescence staining of wild-type and *Gcna*$^{-}$/Y testis sections from 6 months old mice. DNMT3B was displayed in red, PLZF in green and DNA (DAPI stained) in blue. Cells positive for both PLZF and DNMT3B are highlighted in yellow and PLZF only cells are highlighted in white.

(G) Frequency of PLZF positive cells also positive for DNMT3B at 1.5 and 6 months old. A minimum of 50 PLZF positive cells are scored per mouse. Data represent the mean and S.D. (n=3 mice for each genotype and age). alues ere calculated using an unpaired t test.

Supplementary Figure S7. Mouse GCNA does not bind DNA *in vitro*.

(A) A DNA pulldown was performed with recombinant FLAG-mGCNA and FLAG-mSPRTN E113Q expressed in a cell-free system. Products of this pulldown were then analysed by Western blot.

(B) Western blot of the DNA pulldown. The blot was probed with an anti-FLAG antibody. Data is representative from two independent experiments.

Supplementary Figure S8. Co-expression of PCNA and GCNA in mouse pre-spermatid cells.

(A) Immunofluorescence staining of wild-type and *Gcna*$^{-}$/Y adult testis sections. PCNA was displayed in red, GCNA (Tra98) in green and DNA (DAPI stained) in blue. Data is representative from three independent experiments.

(B) Frequency of PCNA positive cells co-expressing GCNA per tube. A minimum of 580 cells are scored per mouse. Data represent the mean and S.D. (n=3 mice for each genotype).

(C) Statistical analysis of the correlation between GCNA and PCNA calculated using GraphPad Prism.

Supplementary Figure S9. DNA breaks in USGs of 6 weeks old mice.

(**A**) Immunofluorescence staining of wild-type and *Gcna*$^{-}$/Y testis sections from 6 weeks old mice. γH2AX was displayed in red, PLZF in green and DNA (DAPI stained) in blue. Cells positive for both PLZF and γH2AX are highlighted in yellow and PLZF only cells are highlighted in white.

(**B**) Frequency of PLZF positive cells also positive for γH2AX at 6 weeks old. A minimum of 50 PLZF positive cells are scored per mouse. Data represent the mean and S.D. (n=3 mice for each genotype). P values were calculated by using an unpaired t-test.

(**C**) Quantification of the amount of fluorescence (integrated density) of γH2AX per USG, in wild-type and *Gcna*$^{-}$/Y testis from 6 weeks old mice. Wild-type (n=179 USGs from three mice), *Gcna*$^{-}$/Y (n=178 USGs from three mice). Data represent the median and interquartile range. P values were calculated by using a two-tailed Mann-Whitney test.

Supplementary Figure S10. Generation and validation of GCNA-deficient mESC.

(A) Validation of the mouse ES cell lines by PCR, by using the PCR primers showed in Supplementary Figure S1.

(B) Validation of the mouse ES cell lines by Western blot. Blot was probed with an antibody directed against mouse GCNA (GCNA-1), with an anti-Nanog antibody and an anti-tubulin antibody.

(C) Micrographs of the mouse ES cell lines in culture.

(D) Representative 2D cell cycle flow cytometry plots of wildtype and GCNA deficient mESCs.

(E) Quantification of the distribution of cells at each stage of the cell cycle in both wild type and GCNA deficient mESCs. Data represent the mean and the S.D. (n=3 independent experiments).

Supplementary Figure S11. Mouse GCNA is related to the Intrasically Disordered Region (IDR) of human GCNA.

(**A**) Schematic representation of the annotated mouse GCNA protein. Mouse GCNA is fully disordered and possess multiple repeats domains. Among them, two are highly acidic (red) and two are basic (blue). SUMO-interacting motifs are represented in light green. A serine-rich region is represented in dark green.

(**B**) Western blot of GCNA from different sources. This Western blot displays extracts from wild-type and *Gcna*$^{-}$/Y testes, wild-type and *Gcna*$^{-}$/Y mESCs and *E.coli* transformed with an expression plasmid (induction with IPTG). Then, the blot was probed against mouse GCNA (TRA98), a testis marker (SYCP3), a mESC marker (Nanog) and a marker for *E.coli* (GroEL). Data is representative from two independent experiments.

(**C**) Western blot of GCNA from different sources. This Western blot displays extracts from wild-type and *Gcna*$^{-}$/Y testes, wild-type and *Gcna*$^{-}$/Y mESCs and *E.coli* transformed with an expression plasmid (induction with IPTG). Then, the blot was probed against mouse GCNA (GCNA-1), a testis marker (SYCP3), a mESC marker (Nanog) and a marker for *E.coli* (GroEL). Data is representative from two independent experiments.

(**D**) Percentage of negative charges in IDRs of metazoans GCNA proteins. Proteins are distributed according to their percentage of negative charges.

(**E**) Distributions of charges in IDRs of metazoan GCNA proteins. Acidic residues are displayed in red and basic residues in blue. Proteins are distributed according to their percentage of negative charges.

Supplementary Figure S12. Biochemical features of mouse GCNA.

(A) Western blot of the FLAG immunoprecipitation. 3T3 cells were transiently transfected with FLAG-mGCNA and a FLAG immunoprecipitation was performed on the soluble fraction. The blot was probed with anti-FLAG, anti-H1, anti-H3 and anti-beta-actin antibodies. Data is representative from two independent experiments.

Supplementary Figure S13. Purification of recombinant MBP-mGCNA.

(**A**) Scheme of purification strategy.

(**B**) Left panel: Coomassie staining of purified MBP-mGCNA and MBP analysed by SDS-PAGE. Middle and right panels: Western blot analyses of purified MBP-mGCNA and MBP, respectively stained with anti-MBP and anti-GCNA (GCNA-1) antibodies.

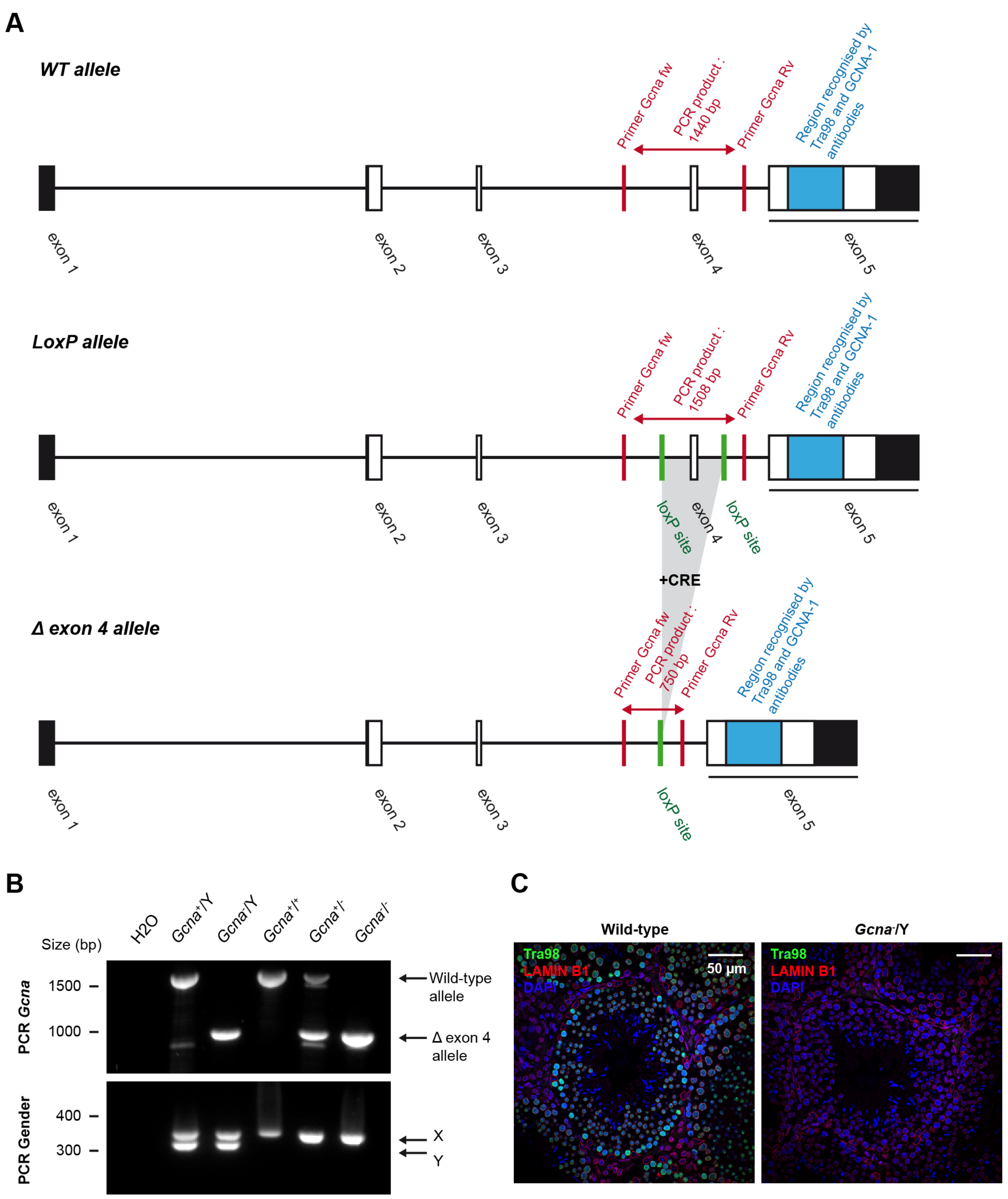

Supplementary Figure S1. Generation and validation of GCNA-deficient mice.

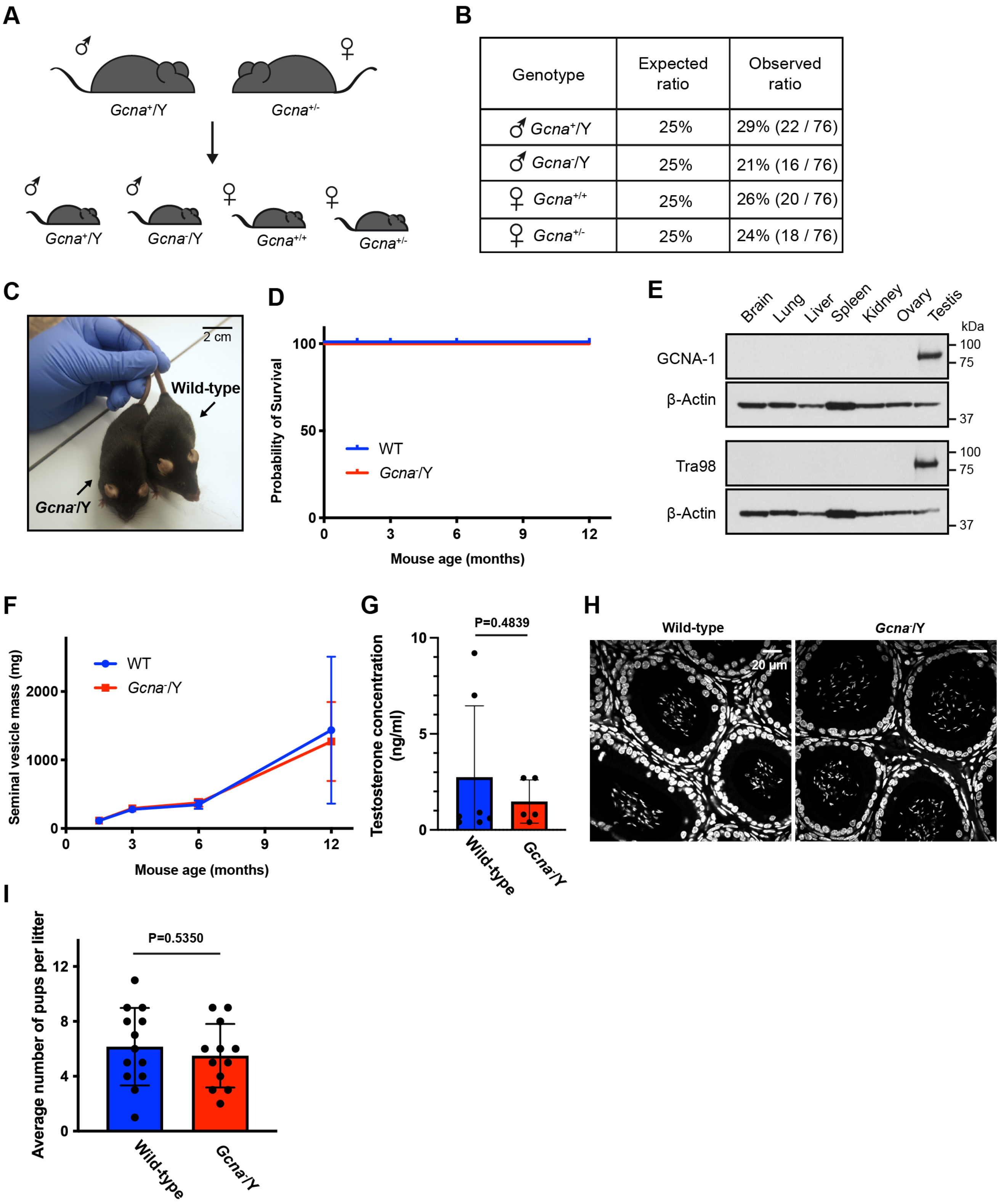

Supplementary Figure S2. Phenotyping of GCNA-deficient male mice.

A

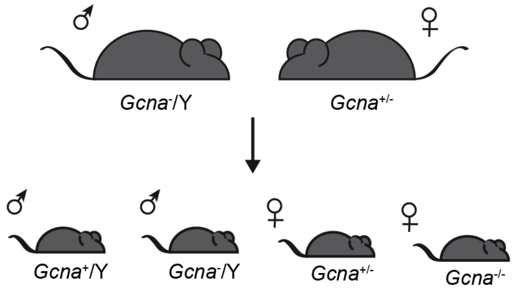

B

Genotype	Expected ratio	Observed ratio
♂ *Gcna*$^{+}$/Y	25%	26% (11 / 43)
♂ *Gcna*$^{-}$/Y	25%	23% (10 / 43)
♀ *Gcna*$^{+/-}$	25%	30% (13 / 43)
♀ *Gcna*$^{-/-}$	25%	21% (9 / 43)

C

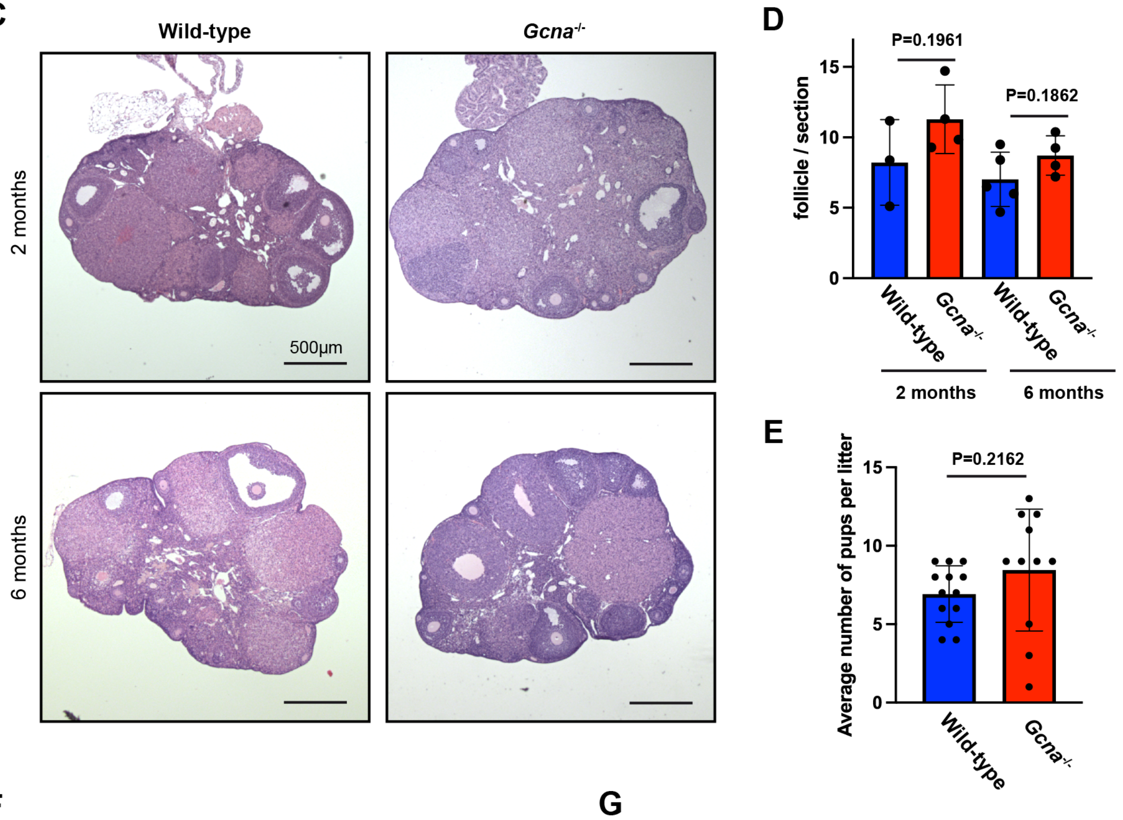

F

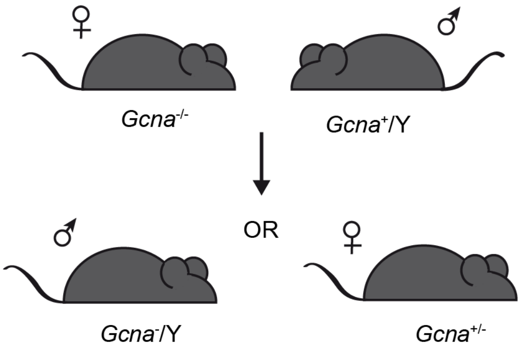

G

Genotype	Expected ratio	Observed ratio
♂ *Gcna*$^{-}$/Y	50%	50% (38 / 76)
♀ *Gcna*$^{+/-}$	50%	50% (38 / 76)

Supplementary Figure S3. Fertility of GCNA-deficient female mice.

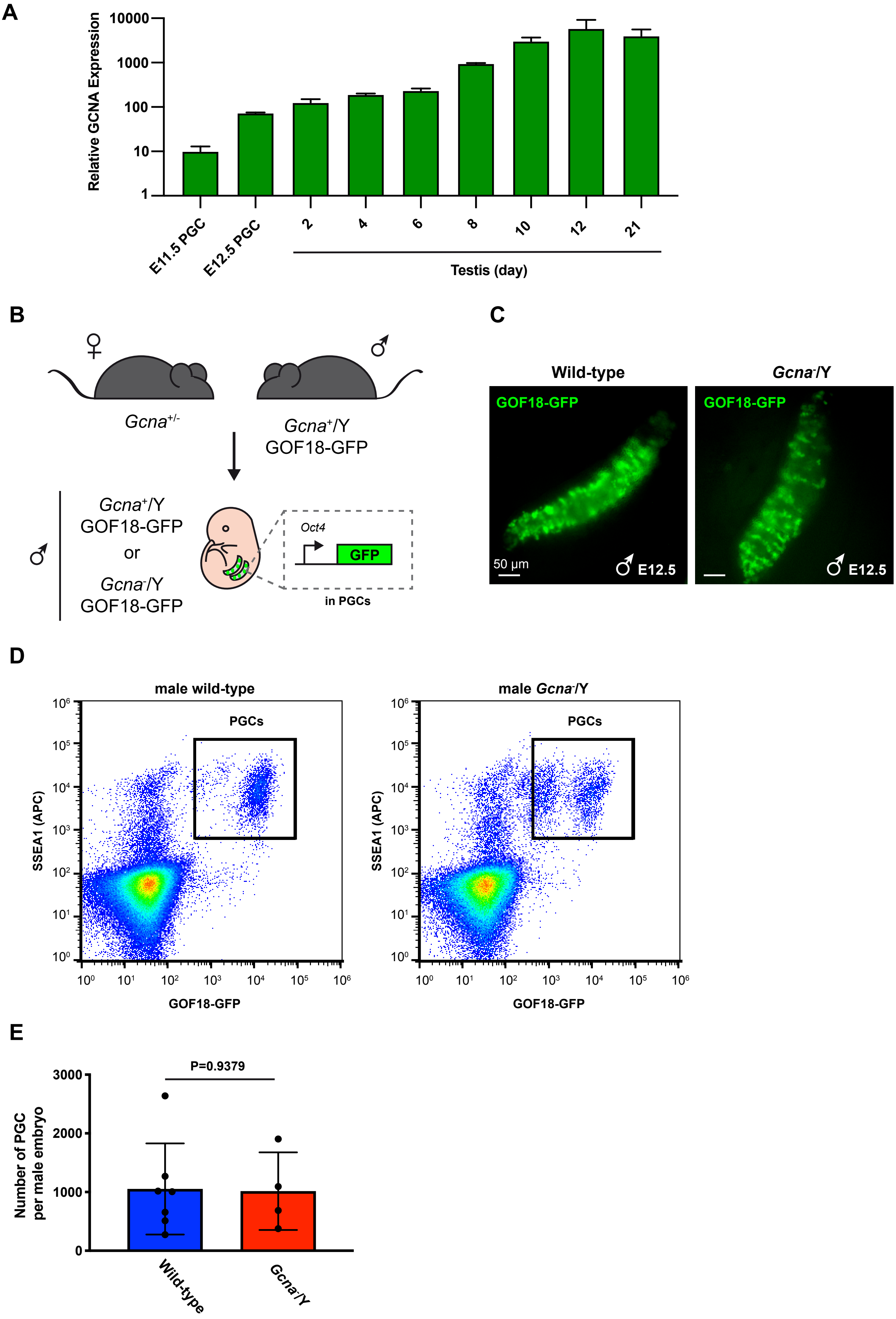

Supplementary Figure S4. GCNA is dispensable for male PGC development.

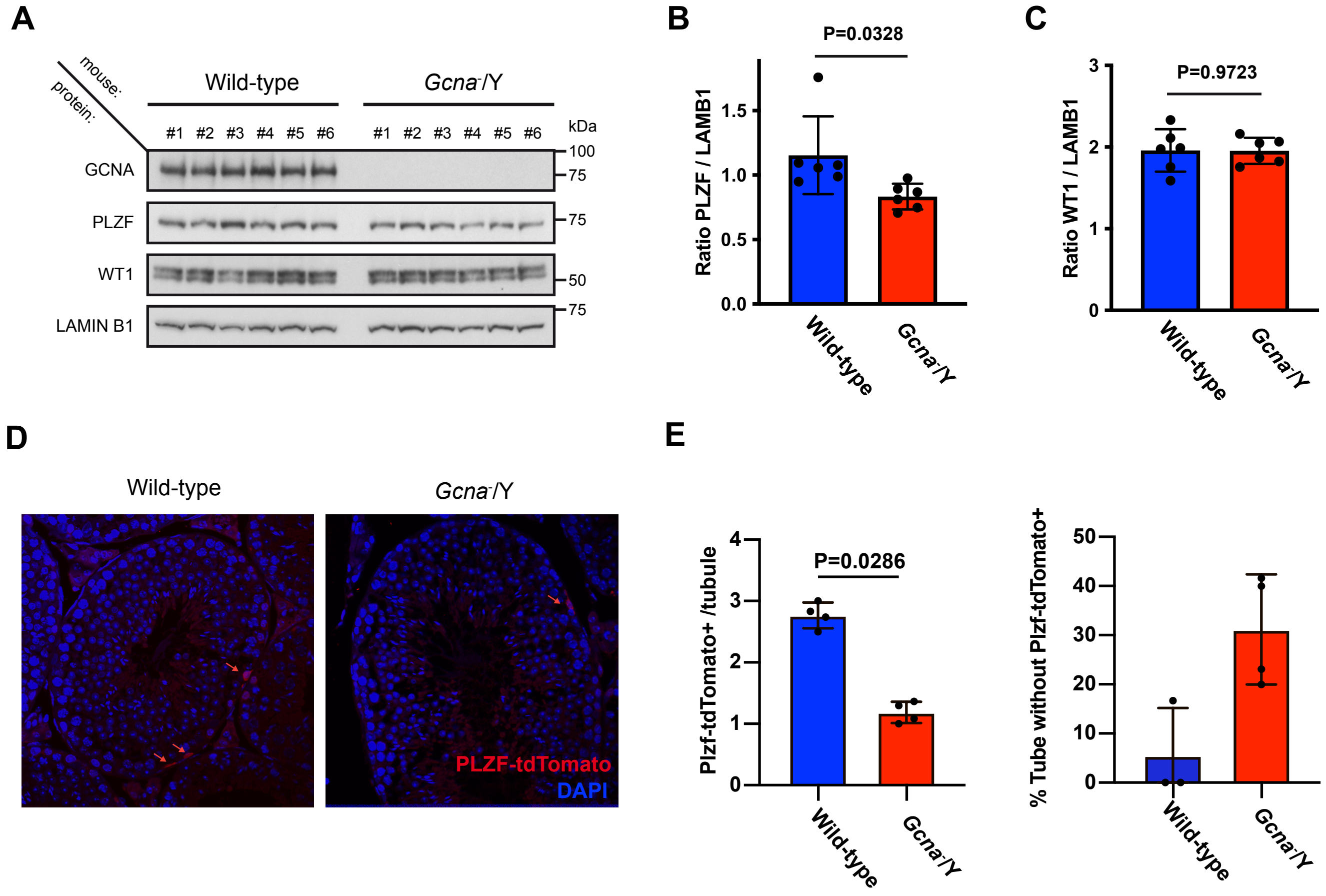

Supplementary Figure S5. GCNA is required for maintenance of PLZF+ cells

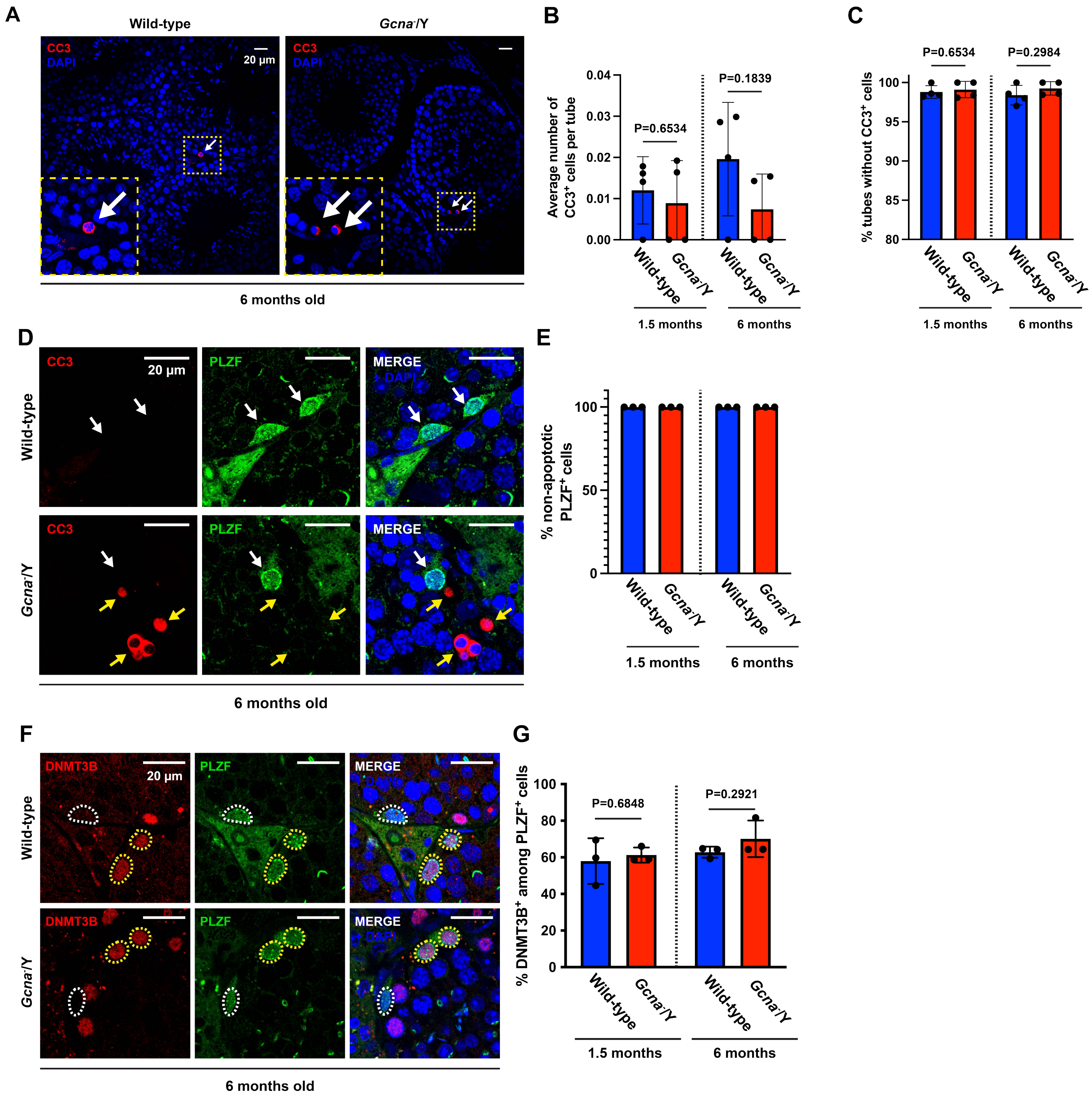

Supplementary Figure S6. USGs are not exhibiting increased apoptosis nor enhanced differentiation in absence of GCNA.

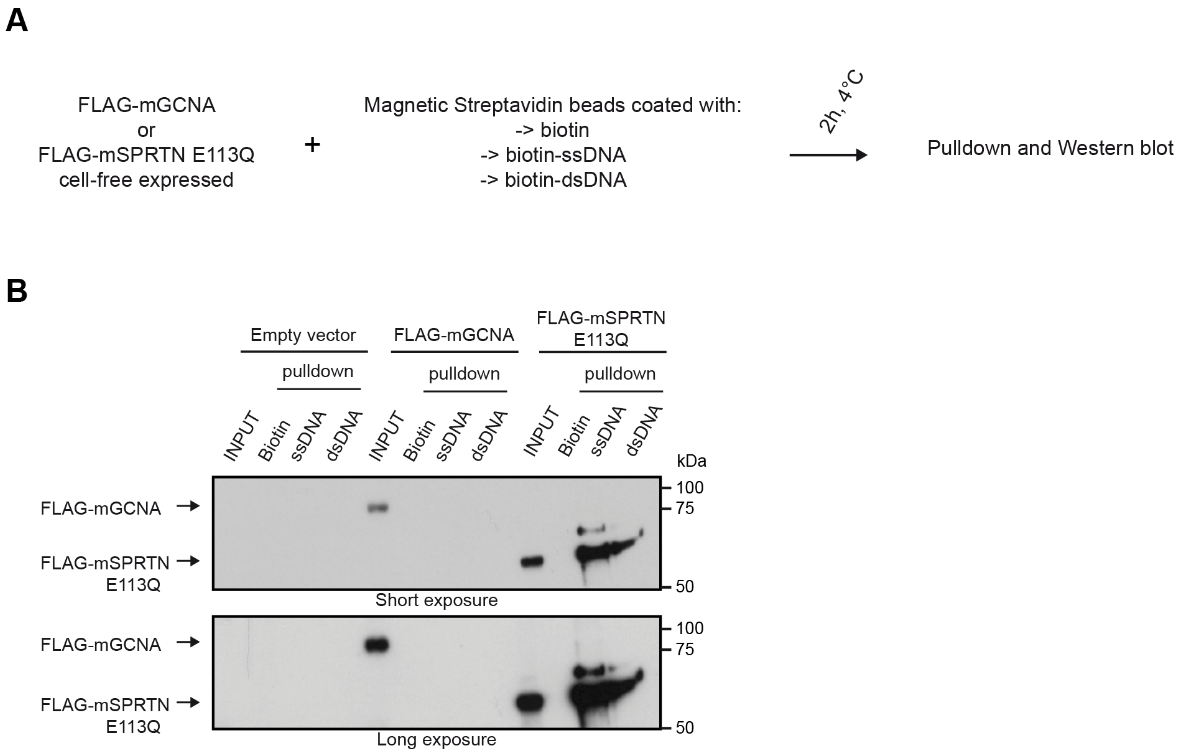

Supplementary Figure S7. Mouse GCNA does not bind DNA in vitro.

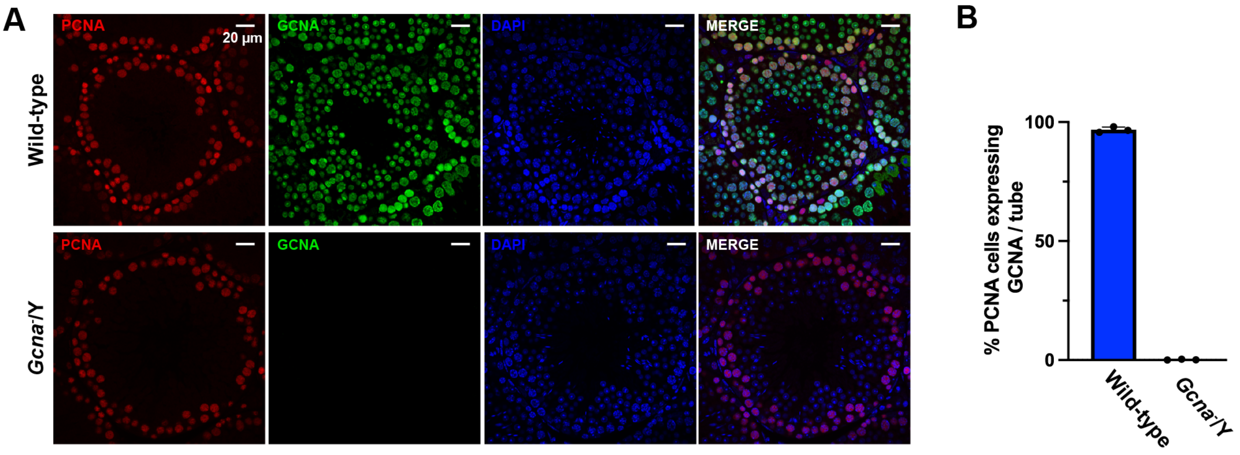

C

Pearson r parameters	
r	0.4532
95% confidence interval	0.3711 to 0.5284
R squared	0.2054
**P value parameters**	
P (two tailed)	<0.0001
Significant? (alpha = 0.05)	Yes
**Number of GCNA-PCNA Pairs**	394

Value of r	Interpretation
1.0	Perfect correlation
0 to 1	The two variables tend to increase or decrease together.
0.0	The two variables do not vary together at all.
-1 to 0	One variable increases as the other decreases.
-1.0	Perfect negative or inverse correlation.

Supplementary Figure S8. Co-expression of PCNA and GCNA in mouse pre-spermatid cells.

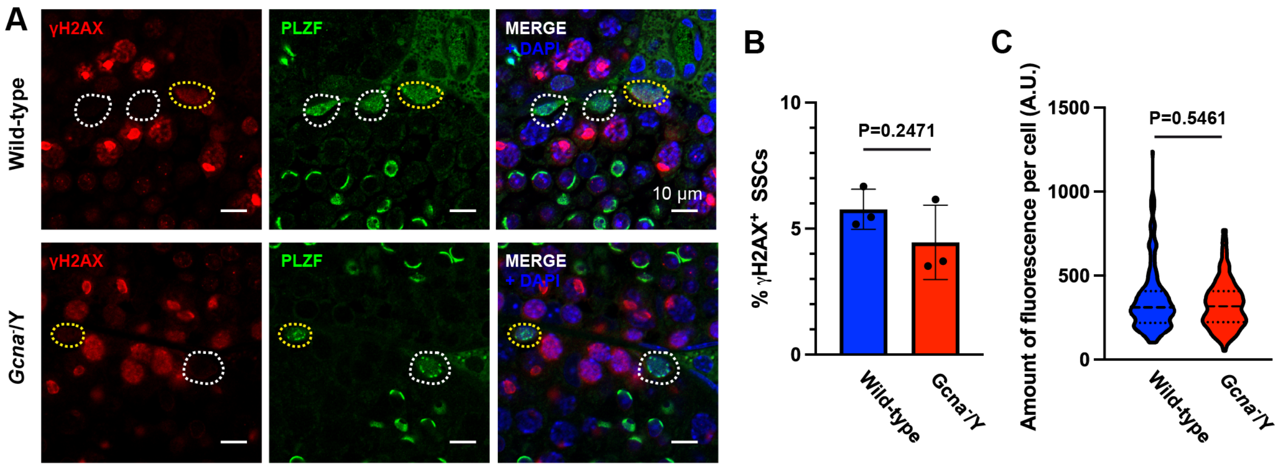

Supplementary Figure S9. DNA breaks in USGs of 6 weeks old mice.

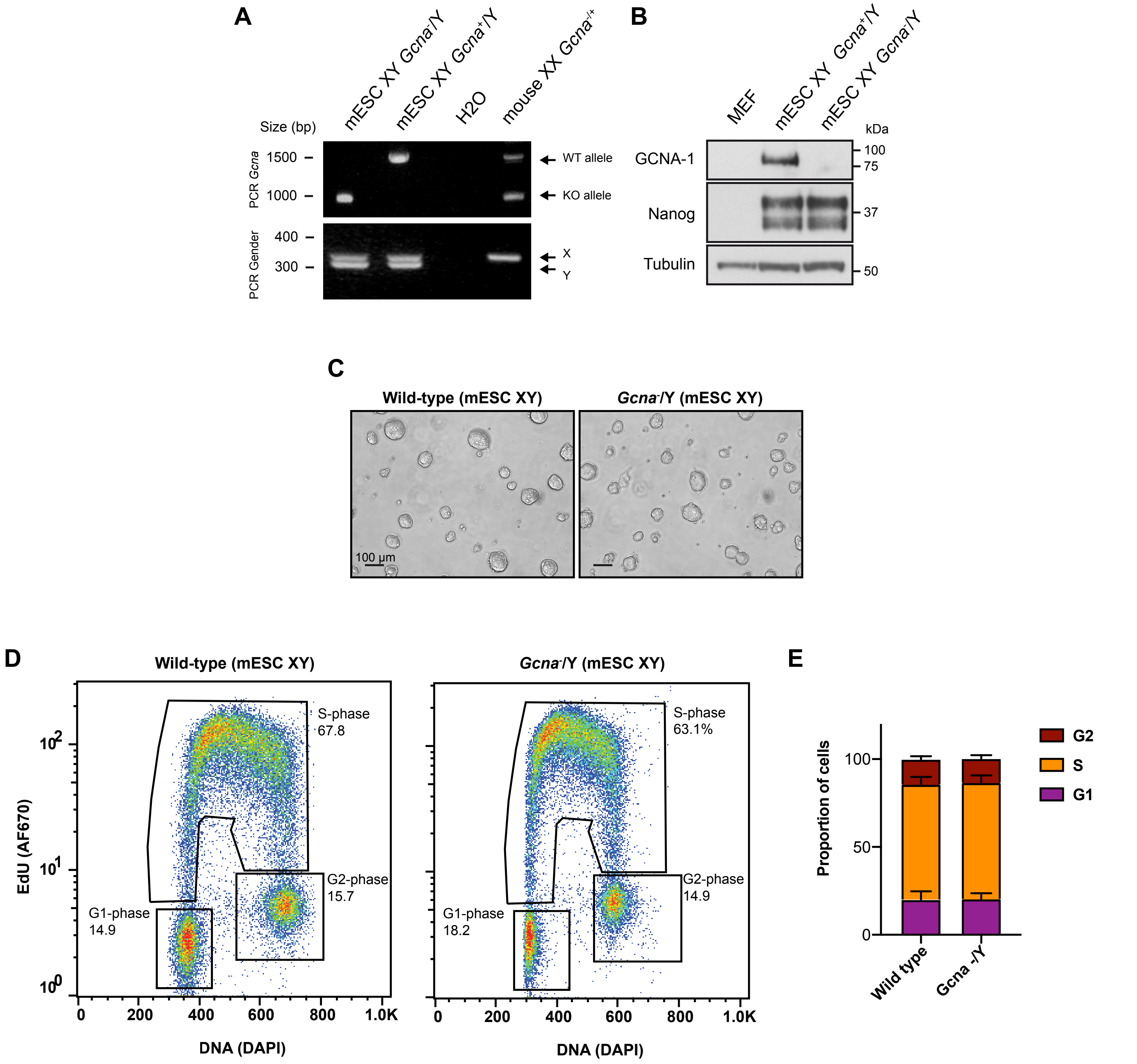

Supplementary Figure S10. Generation and validation of GCNA-deficient mESC.

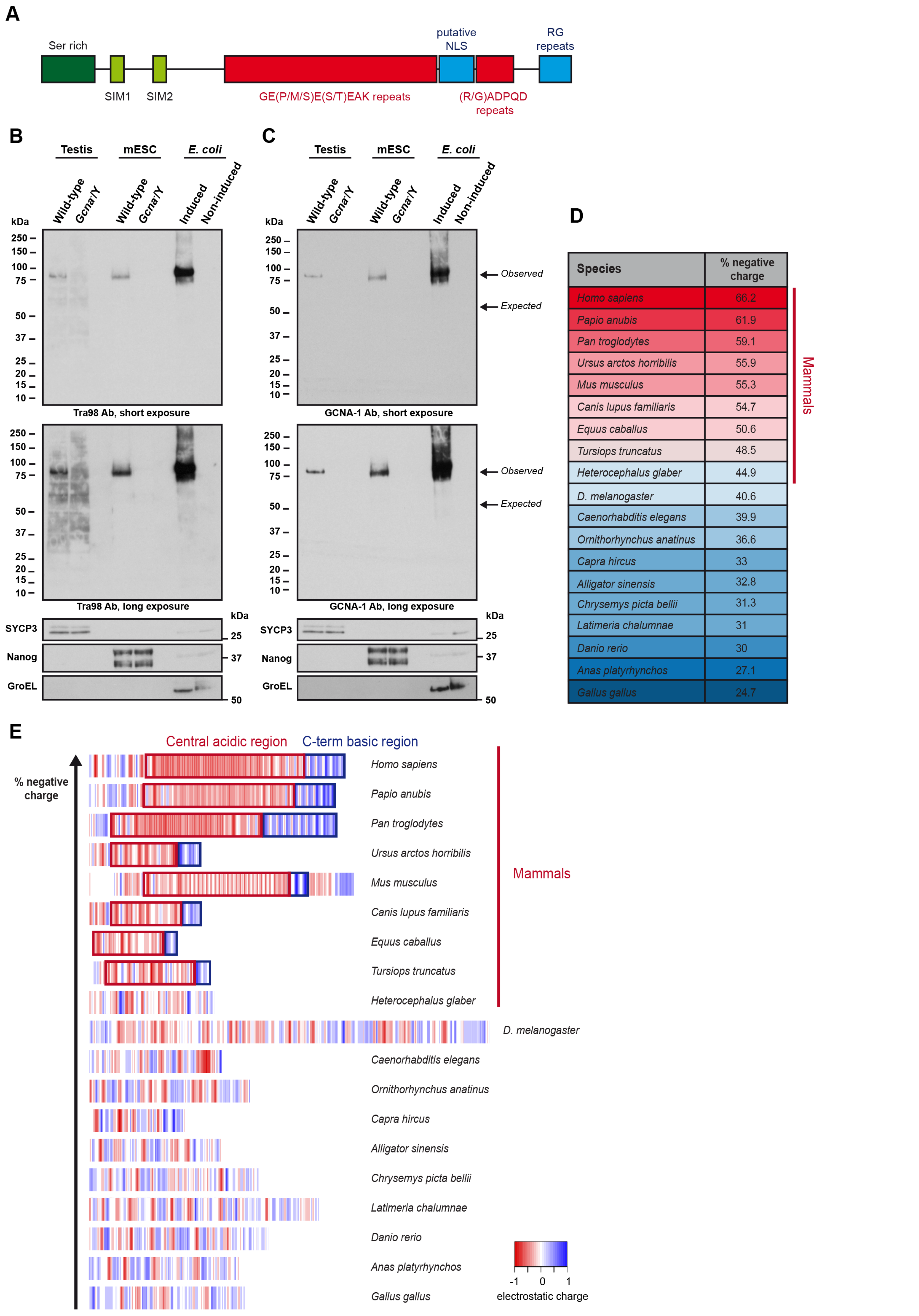

Species	% negative charge
Homo sapiens	66.2
Papio anubis	61.9
Pan troglodytes	59.1
Ursus arctos horribilis	55.9
Mus musculus	55.3
Canis lupus familiaris	54.7
Equus caballus	50.6
Tursiops truncatus	48.5
Heterocephalus glaber	44.9
D. melanogaster	40.6
Caenorhabditis elegans	39.9
Ornithorhynchus anatinus	36.6
Capra hircus	33
Alligator sinensis	32.8
Chrysemys picta bellii	31.3
Latimeria chalumnae	31
Danio rerio	30
Anas platyrhynchos	27.1
Gallus gallus	24.7

Supplementary Figure S11. Mouse GCNA is related to the Intrasically Disordered Region of human GCNA.

A

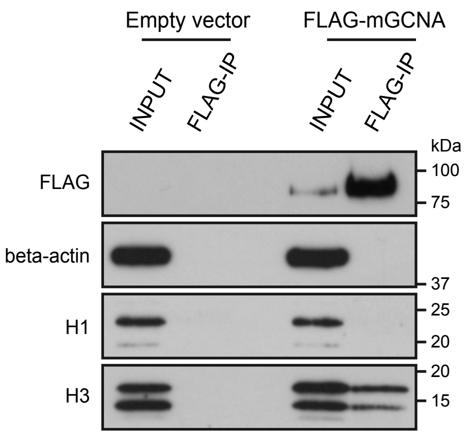

Supplementary Figure S12. Biochemical features of mouse GCNA.

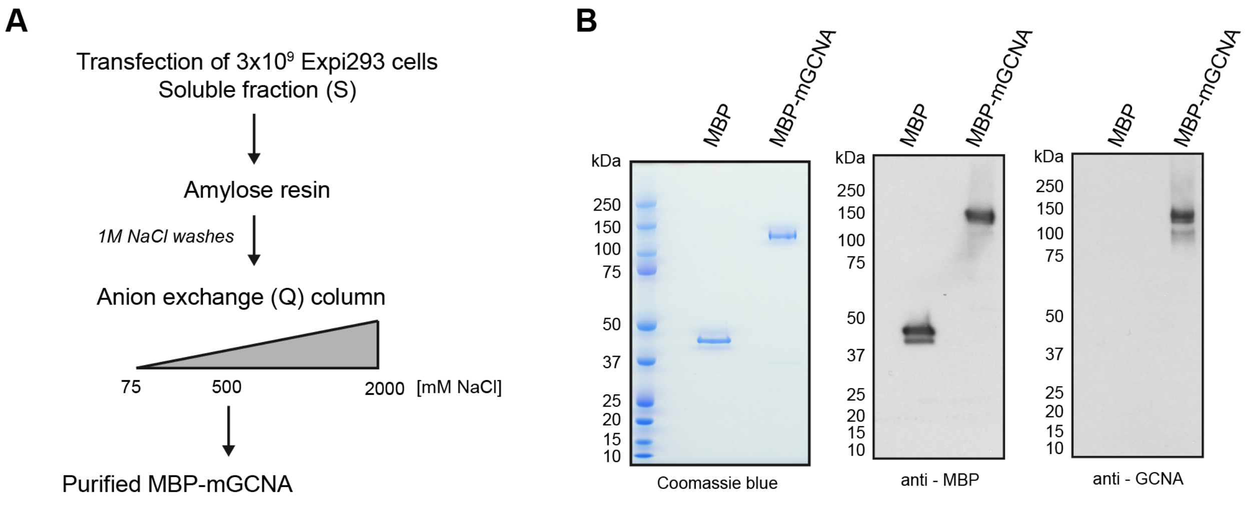

Supplementary Figure S13. Purification of recombinant MBP-mGCNA.
